# Recent contributions in the field of the recombinant expression of disulfide bonded proteins in bacteria

**DOI:** 10.1186/1475-2859-11-129

**Published:** 2012-09-14

**Authors:** Ario de Marco

**Affiliations:** 1 Department of Environmental Sciences, University of Nova Gorica, Vipavska 13, Rozna Dolina (Nova Gorica), PO Box301, SI-5000, Slovenia

## Abstract

The production of heterologous disulfide bonded proteins in bacteria remains a biotechnological challenge. A rapid literature survey results in the identification of some interesting proposals, such as the option of producing functional proteins in the cytoplasm in the presence of sulfhydryl oxidases and isomerases. Furthermore, an ever-increasing number of applications refers to recombinant proteins displayed at the bacterial surface. Time will tell whether these developments will lead to universally accepted laboratory protocols.

## Circumventing the obstacle

The time is ripe for an update on the innovations introduced for an effective bacterial production of heterologous polypeptides that require the formation of disulfide bonds in order to reach their stable native conformation [1]. Over the last three years, Microbial Cell Factories (MCF) has been very alert to the developments in the field and published articles that are representative of the alternative strategies proposed to circumvent the objective reluctance of bacteria to produce this class of proteins. It is possible to group them according to the proposed approach.

a) Be fit: optimize the conditions

Trivial as it may sound, optimized cell factories are more efficient than unsuitable bacteria, but what features make them perform better? For instance, it is known that recombinant expression in bacteria can be substantially improved by the co-expression of molecular foldases and the addition of osmolytes, albeit the outcome is extremely protein-dependent [1]. From this perspective, the data that correlate for the first time the stabilizing effects of different chemical chaperones to specific molecular features of the target proteins are very interesting and open the possibility of predicting the optimal mix for any given polypeptidic sequence to be expressed [2]. Another approach considers the preparation of large collections of mutants covering the whole genome and that allow for the selection of *ad hoc* strains with improved capacities for extremely specialized metabolic tasks [3-6]. Nevertheless, it is necessary to bear in mind that the expression strain represents only one of the production factors and that codon optimization and the co-expression of stabilizing factors can be critical for the goal accomplishment [7,8].

b) In the periplasm, but better than ever

Recombinant disulfide bonded proteins have been preferentially produced in the bacterial periplasmic space because of its favourable redox conditions. However, the method is notorious for resulting in low yields and proteins that often fail to fold correctly [1]. Recently, the work of Ow et al. [9] has shown that protein misfolding and aggregation in the periplasm reduced significantly the cell viability and that overexpressing the periplasmic chaperones Skp or FkpA could reverse both these shortcomings. Yields can be increased and purification procedures improved also by selecting the most suitable leader peptide. For instance, the domain D of protein A allows for the stabilization, translocation and straight-forward purification of fused proteins [10], whereas SRP leader peptides were more effective than SEC ones for secreting recombinant antibodies with very favourable thermodynamic and that started being partially folded in the cytoplasm. Since linearization is a strict requisite for polypeptide transfer into the periplasm, antibodies bound to the SEC secretion route were trapped in the cytoplasm because they began folding after synthesis while the simultaneity of synthesis and translocation assured by the SRP pathway resulted in higher yields [11].

c) Ejected in the All: harnessing the secretion pathways

Bacteria have the capacity of secreting proteins into the external medium and this alternative has been evaluated in the past for biotechnological applications [1]. The main advantages are: i) oxidizing environment; ii) avoiding of saturation due to the large available volume; iii) simplified protein purification due to the low amount of contaminants. Extracellular release can be also exploited for therapeutic aims, as it is the case of an anti-trypanosoma recombinant antibody expressed in the symbiont bacteria (*Sodalis glossinidius*) of tsetse fly [12]. Although the secretion mechanisms are not always well characterized [13], the systems set for accumulating the target proteins in the cell supernatant never seemed as popular as recently. *E. coli* alpha-hemolysin type I and autotransporter secretion systems [14,15], *Salmonella* flagellar type III [16] as well as the combinations leading to target protein cell display in different bacteria [17-22] underline the present available methodological variety. In particular, the display systems combined to flow-cytometry and magnetic cell sorting seem to be very promising for large-scale screenings of polypeptides with desired characteristics. In such a way, antigenic and curative peptides to be used for vaccination have been identified [22] and *E. coli* displaying both *Mycobacterium tuberculosis* and *Salmonella enterica* vaccine targets have been foreseen as live vaccines [18,20]. Finally, bacteria displaying recombinant antibodies were directly spotted on chips for preparing effective diagnostic microarrays without the necessity of any purification step [23].

d) Inverting the paradigm: a new perspective for cytoplasmic production

The work began twenty years ago for elucidating the mechanisms regulating the redox conditions in bacteria [1,24] finally resulted in the commercialization of different strains with diminished cytoplasmic reductive pathways. SHuffle is the last proposal in the field, a strain that combine improved oxidative conditions with the cytoplasmic expression of the DsbC isomerase [25]. As it is (always) the case, the success of this strain will remain construct-dependent, as illustrated by the deceiving results obtained when it was compared to Origami 2 [26] or tested for the cytoplasmic expression of *Metarhizium anisopliae*, a protein for which the fusion to the MBP-tag and the chaperoning features of DsbC were extremely more profitable [27]. Two papers from Ruddock’s group [28,29] probably represent the real break-through concerning the production of disulfide-bond-dependent proteins in bacterial cytoplasm. For the first time, it was demonstrated that disulfide bonds could be correctly formed in this cell compartment without modifying the redox conditions, but simply forcing the disulfide bond formation in the presence of over expressed sulfhydryl oxidase and disulfide-bond isomerases. In this way, the cell metabolism is not compromised as it is the case in oxidizing strains [26] and enables yields that have never been obtained in mutant strains with reducing cytoplasm or when only the restricted periplasmic space is available for recombinant protein accumulation. This approach has already successfully repeated in several independent labs [30,31], a promising indication that it may represent a general reliable method for protein production.

## Conclusions

Recombinant protein production is still far from being a mature discipline and the innovative contributions briefly listed in this compendium show clearly that the platform constantly moves forward. Some tactics, such as secretion strategies and combinatorial approaches, seem to gain attention whereas other methods, such as refolding from inclusion bodies [32], have not significantly developed lately. The scope of this short update is to show tendencies rather than to add another exhaustive review to the list and at MCF we are interested in the feedback of our readers concerning this form of communication.
